# Munronoid I Ameliorates DSS-Induced Mouse Colitis by Inhibiting NLRP3 Inflammasome Activation and Pyroptosis *Via* Modulation of NLRP3

**DOI:** 10.3389/fimmu.2022.853194

**Published:** 2022-07-05

**Authors:** Xingyu Ma, Qianqian Di, Xiaoli Li, Xibao Zhao, Ruihan Zhang, Yue Xiao, Xunwei Li, Han Wu, Haimei Tang, Jiazheng Quan, Zherui Wu, Weilie Xiao, Weilin Chen

**Affiliations:** ^1^ Marshall Laboratory of Biomedical Engineering, Department of Immunology, Shenzhen University School of Medicine, Shenzhen, China; ^2^ Key Laboratory of Medicinal Chemistry for Natural Resource, Ministry of Education, Yunnan Research & Development Center for Natural Products, School of Chemical Science and Technology, Yunnan University, Kunming, China

**Keywords:** inflammatory bowel diseases, NLRP3: NOD-like receptor pyrin domain-containing protein 3, Munronoid I, pyroptosis, ubiquitination

## Abstract

Inflammatory bowel diseases (IBDs) are increasingly common diseases characterized by chronic and relapsing inflammation of the gastrointestinal tract. NLRP3 might be a crucial regulator of the homeostatic balance of the intestine, but its upregulation leads to pyroptosis. Munronoid I is extracted and purified from *Munronia sinica*, which has shown an anti-inflammatory effect, but the efficacy of Munronoid I in IBD remains unproven. In this study, we attempted to determine the effect of Munronoid I on NLRP3 to regulate the inflammasome activation and pyroptosis in IBD. Our data demonstrated that Munronoid I treatment attenuated DSS-induced body weight loss, pathological injury of the colon, the production of IL-1β and IL-18, and the expression of pyroptosis-associated proteins in colon tissue in mice. Moreover, Munronoid I inhibited LPS/ATP-induced pyroptosis in mouse peritoneal macrophages, MODE-K cells, and DSS-induced pyroptosis in mouse colonic epithelial cells, and decreased the release of inflammatory cytokines IL-1β and IL-18 in mouse peritoneal macrophages. Mechanically, Munronoid I could suppress the NLRP3 inflammasome activation and pyroptosis by promoting the K48-linked ubiquitination and NLRP3 degradation. It is suggested that Munronoid I might be a potential therapeutic candidate for IBD.

## Introduction

Inflammatory bowel diseases (IBD) are chronic and relapsing diseases of the gastrointestinal tract, including Crohn’s disease (CD) and ulcerative colitis (UC) (1–3). The incidence of IBD is high in Western countries. Recent studies have shown that the incidence is increasing in Asia, Africa, and South America (4–6). Although the incidence in China is low, the number of people suffering from IBD is one of the highest in the world due to the large population. The etiology of IBD is complex, including unknown environmental stimuli (7, 8), diet (9), family genetics (10, 11), uncontrolled immune responses (12, 13), and intestinal microbial disorders (14, 15). There is currently no clinically effective treatment for IBD, and pharmacological treatment is mainly with immunosuppressive drugs and broad-spectrum antibiotics, which have relatively high side effects (16, 17). Due to side effects such as the risk of serious infections, metabolic disorders, and organ toxicity caused by immunosuppressants (18, 19), finding new therapies with fewer side effects has become a future strategy for the treatment of IBD.

By definition, pyroptosis is a pro-inflammatory programmed cell death that depends on the activation of inflammatory cysteine-dependent aspartate-specific proteases (caspase) (20, 21). Previous studies have reported that there are two pathways to induce pyroptosis, namely the canonical pathway and the non-canonical pathway (22, 23). The canonical pathway: extracellular lipopolysaccharide (LPS) initiated the activation of NOD-like receptor pyrin domain-containing protein 3 (NLRP3) inflammasome priming phase (24), and adenosine triphosphate (ATP) promoted the assembly and activation of NLRP3 inflammasome (25), followed inducing cell death by pyroptosis. NLRP3 is a protein consisting of a C-terminal leucine-rich repeat (LRR) domain, nucleotide-binding and oligomerization domain (NACHT), pyrin domain (PYD) (26), which interacts with apoptosis-associated speck-like proteins containing a caspase-recruitment (CARD) domain (ASC) by PYD (27). Adaptor protein ASC recruits procaspase-1 by CARD domain to assemble into NLRP3 inflammasome (28, 29), after procaspase-1 shears itself into a mature caspase-1 p20 and p10 subunits (30), activated caspase-1 cleaves inflammatory cytokines pro-interleukin 1 beta (pro-IL-1β), pro-IL-18 (31), and gasdermin D (GSDMD) to promote pyroptosis (32). The non-canonical pathway: intracellular LPS directly recognized by procaspase-11 (in mouse) or procaspase-4/5 (in human) (33), then self-shear activation as procaspase-1, activated caspase-11, or caspase-4/5 cleaves GSDMD but does not cleave pro-IL-1β, pro-IL-18 (34). The cut GSDMD perforates the cell membrane leading to pyroptosis (32, 35). NLRP3 inflammasomes are emerging as key regulators of the innate immune response, and several studies have demonstrated that the activity of the inflammasome was closely related to IBD (36–38). Therefore, NLRP3 may be a potential therapeutic target for the treatment of IBD.

The mechanism by which inflammatory caspases promote pyroptosis has long been unknown, and recent pyroptosis studies have identified a substrate of caspase-1 and caspase-4/5/11 as the executor of pyroptosis, known as GSDMD (32). Either in the canonical pathway or non-canonical pathway, GSDMD has high performance in the process of pyroptosis (32, 35). GSDMD consists of 480 amino acids and is divided into two domains, the C-terminal repressor domain (p20) and the N-terminal pore-forming domain (p30), respectively (39). GSDMD p20 subunit suppresses the activation of the p30 subunit, resulting in the intact protein being inactive once cleaved by caspase-1 and caspase-4/5/11, the two subunits are separated, and the activated p30 subunit clusters to form inner diameters of 10–16 nm pores in the cell membrane (39, 40). The inflammatory cytokines IL-1β and IL-18 can be secreted from these pores into the extracellular matrix (39, 41), besides, the influx of Na^+^ ions into the cell causes an increase in intracellular osmotic pressure (42–44), resulting in a large amount of water entering the cell and causing it to burst (45) the cell pyroptosis and eventually amplifying the inflammatory response. The other members of the gasdermin family, GSDMA, GSDMB, GSDMC, and GSDME, also have pore-forming activity with their N-terminal pyroptosis-inducing domain (39, 46). Studies have shown that caspase-3 specifically cleaved GSDME and thereby induces pyroptosis (47).

**Figure 1:**
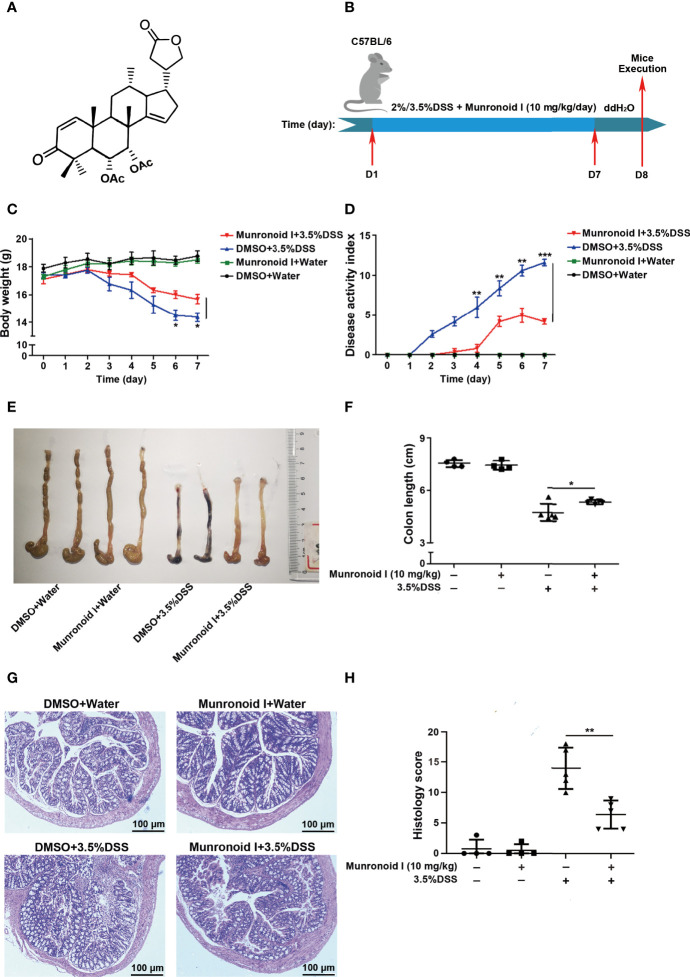
Munronoid I relieves the severity of DSS-induced colitis in mice. **(A)** The chemical structure of Munronoid **(I) (B)** Overview of the experimental protocol. C57BL/6 mice (n = 4–5 mice/group) were fed with distilled water or water containing 3.5% DSS and were intragastric injected with DMSO or Munronoid I (10 mg/kg) daily. Seven days later, all the water was changed to distilled water, on the 8th day, the mice were sacrificed, and their colon tissue was collected. **(C, D)** The body weight **(C)**, stool consistency, and gross rectal bleeding were recorded every day to calculate the DAI score **(D)**. **(E)** Colons of mice were assessed at the time of necropsy with representative macroscopic images. **(F)** The colon length was measured and statistical significance determined by unpaired Student’s t-test. **(G)** H&E staining of colon sections (10X) and representative H&E images scored in **(H)**. Data in C, D, F and H presented as the mean ± SD. *p<0.05, **p<0.01, ***p<0.001.

Munronoid I is a novel diterpenoid compound (48), which was extracted and purified from *Meliaceae* family, and its structure is shown in **Figure 1A**, with the molecular formula of C_20_H_18_O_5_ and molecular weight of 512 Da. Some previous studies have indicated that Munronoid I might have anti-neoplastic and anti-inflammation activities (49, 50), but no studies have shown its effect on pyroptosis. In the present study, we demonstrated for the first time that Munronoid I has a protective effect in a murine model of IBD. This study showed that administration of Munronoid I remarkably reduced the inflammatory cells infiltration in the colon, loss of weight, disease activity index (DAI), and the production of pyroptosis-related protein in colon issue of colitis mice. Further, Munronoid I significantly diminished LPS and ATP-induced pyroptosis and IL-1β and IL-18 production in mouse peritoneal macrophages *in vitro*. In addition, Munronoid I downregulates the expression of NLRP3 by promoting K48-linked ubiquitination and NLRP3 degradation. Thereby, Munronoid I may be a potential therapeutic candidate for IBD and pyroptosis-related disease.

## Materials and Methods

### Reagents and Antibodies

Dextran sulfate sodium salt (DSS) was purchased from MP Biomedicals (CA, USA), adenosine triphosphate (ATP) was obtained from *In vivo*Gen Biotechnology (CA, USA), Methyl sulfoxide (DMSO), lipopolysaccharide (LPS), and thioglycolate were purchased from Sigma-Aldrich (MO, USA). Carbobenzoxy-Leu-Leu-leucinal (MG132), chloroquine (CQ), and cycloheximide (CHX) were purchased from Selleck (TX, USA). Dithiothreitol (DTT), ethylene diamine tetraacetic acid (EDTA), and primers used in this study were synthesized by Sangon Biotech (Shanghai, China). Antibodies against NLRP3, Caspase-1, and ASC were purchased from Adipogen Life Sciences (CA, USA). Antibody specific to IL-1β was obtained from Cell Signaling Technology. GSDMD antibody was purchased from Abcam (Cambridge, UK). The Flag-tag antibody was obtained from Genscript (Nanjing, China). β-actin and HA-tag antibodies were purchased from Proteintech (Wuhan, China).

### Munronoid I

The compound Munronoid I was extracted from the aerial parts of *Munronia sinica*, and the isolation method was reported according to the reference (49).

### Mice and Cell Cultures

Wild type C57BL/6 mice (female, 6–8 weeks), weighing 16–20 g, were purchased from Vital River Company (Beijing, China). Mice were housed in individual and specific pathogen-free conditions. All animal care and experimental procedures were carried out according to protocols approved by the Animal Care and Use Committee of Shenzhen University School of Medicine (Approval Number: AEWC-2019011) and were in compliance with the Guidelines on Animal Welfare of Shenzhen University School of Medicine.

Mouse peritoneal macrophages and mouse bone marrow derived macrophages (BMDMs) were obtained from C57BL/6 mice and incubated with RPMI 1640. Mouse intestinal epithelial cells MODE-K and HEK293T were cultured in RPMI 1640 and DMEM added with 10% fetal bovine serum (FBS) and antibiotics respectively.

### DSS-Induced Mouse Colitis Model

For the mouse colitis model, C57BL/6 mice were treated with drinking water containing 3.5% DSS or not for 7 days and followed DMSO or Munronoid I (10mg/kg) treatment by gavage daily. After 7 days, the drinking water was switched to normal water and the mice were sacrificed on the eighth day. Colon tissue was collected for subsequent analysis.

### Histopathological Analysis

Formalin-fixed and paraffin-embedded colon tissue was sectioned and stained with hematoxylin and eosin (H&E). A modified version of the scoring system according to a previously published grading system was used (51). The histology score was determined by multiplying the percent involvement of each of the five histological subscores. For each parameter (0, absent; 1, mild; 2, moderate; 3, severe): mononuclear cell infiltrate (0-3), epithelial injury/erosion (0-3), crypt hyperplasia (0-3), polymorphonuclear cell infiltrates (0-3), and transmural inflammation (0-4). Extent factor was derived according to percent area involvement: 0, 0%, 1, < 10%, 2, 10-25%, 3, 25-50%, and 4, >50%.

### Assessment of Disease Activity Index

To assess the severity of colitis, we recorded and calculated the body weight, stool consistency, and gross rectal bleeding according to a previously published grading system (52). Concisely, weight loss: 0–1%, score 0; 1–5%, score 1; 5–10%, score 2; 10–20%, score 3; >20%, score 4. Stool consistency: normal, score 0; loose stools, score 2; watery diarrhea, score 4. Gross rectal bleeding: none, score 0; slight bleeding, score 2; gross bleeding, score 4.

### Preparation of Mouse Peritoneal Macrophages and Munronoid I Treatment

C57BL/6 mice were intraperitoneally injected with 2 ml of 3% thioglycolate broth. On the 4th day, the mice were sacrificed and mouse peritoneal macrophages were collected and isolated by washing three times with PBS. The collected cells were cultured in RPMI 1640 with 10% FBS at 37°C with 5% CO2. Mouse peritoneal macrophages were plated in 12-well plates (1×10^6^ per well) overnight. The cells were pre-treated with DMSO or Munronoid I (0-50 μM) for 2 h, then LPS (100 ng/ml) stimulated for 4 h, and treated with ATP (5 mM) for 30 min. Cell culture supernatants and the protein were collected and stored in -80°C or detected for further analysis.

### Preparation of Mouse Bone Marrow Derived Macrophages and LPS Transfection

Mouse BMDMs were obtained from C57BL/6 mice. Cleaned femurs and tibias were cut and flushed with sterile PBS twice to collect bone marrow cells. The collected bone marrow suspension was cultured with red blood cell lysis buffer for 2 min and then washed with PBS. The collected cells were plated in RPMI 1640 supplemented with 10% FBS and 10 ng/ml macrophage colony-stimulating factor (M-CSF) (Peprotech Inc, NJ, USA) at 37°C with 5% CO2 for 6 days. BMDMs were incubated with DMSO or Munronoid I (50 μM) for 2 h, then poly I:C incubated for 4 h, LPS (2 μg/ml) was transfected by FuGENE (promega, WI, USA) for 16 h. The supernatants and protein were collected for further study.

### Cell Viability Assay and Median Cytotoxic Concentration (CC50) Assay

The cell viability was detected by a CCK-8 assay. Mouse peritoneal macrophages or MODE-K cells were collected and plated in 96-well plates at 1×10^5^ per well overnight. Cells were treated with DMSO or different concentrations of Munronoid I (0–50 µM) for 24 h, and then 10 μl CCK-8 working solution were added to each well and incubated for 2 h at 37°C, according to the manufacturer’s instructions (Yeasen, Shanghai, China). Thereafter, the optical density of each well was recorded at 450 nm using a microplate reader. CC50 of Munronoid I on mouse peritoneal macrophages were also detected by CCK-8 assay. Mouse peritoneal macrophages were treated with DMSO or different concentrations of Munronoid I (0–640 µM) for 24 h, and then 10 μl CCK-8 working solution were incubated for 2 h at 37°C. Thereafter, the optical density of each well was recorded at 450 nm using a microplate reader.

### ELISA

IL-1β, IL-6, TNF-α (mouse) ELISA antibody kit (eBioscience Biotechnology, CA, US) and IL-18 (mouse) ELISA antibody kit (MultiSciences (Lianke), Hangzhou, China) were used to determine IL-1β and IL-18 (mouse) levels in culture supernatants, according to the manufacturer’s instructions.

### Lactate Dehydrogenase Assay

LDH activity in cell supernatants was assessed by an LDH assay kit (Beyotime Biotechnology, Shanghai, China). Briefly, mouse peritoneal macrophages or MODE-K (1×10^5^ per well) were seeded in 96-well plates followed by the Munronoid I treatment. We added 10 μl 10× lysis solution for 1 h before adding the working solution to the maximum LDH release control group. Then 60 μl supernatants and 30 μl working solution were added to a new 96-well plate and incubated for 30 min at room temperature away from light on the shaking table. Finally, the absorbance was measured at 490 nm or 492 nm following the manufacturer’s instructions.

### Hochest33342 and PI Double Staining

Mouse peritoneal macrophages were incubated with DMSO or Munronoid I (50 μM) for 2 h, then stimulated with LPS (100 ng/ml) for 4 h. The cells were treated with ATP (5 mM) for 1h, then the cells were washed twice with cold PBS and incubated with Hochest33342 (5 μg/ml; staining for all cells) and propidium iodide (PI, 2 μg/ml; staining for membrane-damaged cells) double staining of Apoptosis and Necrosis assay Kit (Beyotime Biotechnology, Shanghai, China) at 4°C for 20 min. After that, the treated cells were washed once by ice-cold PBS immediately and observed under fluorescence confocal microscopy.

### Isolation of Colonic Epithelial Cells

Colonic epithelial cells were isolated from a section of colon tissue after mice were treated with 3.5% DSS. The colon tissue was washed with Ca2^+^ free PBS to remove feces. And the tissue was cut longitudinally and oscillated in pre-heated 1mM DTT (Ca2^+^ PBS free) for 10 min at 37°C. Then, the supernatant was taken, the tissues were washed with Ca2^+^ free PBS, and incubated with 1.5mM EDTA (Ca2^+^ free PBS) for 15 min at 37°C. The tissue was then vortexed for 2 min to disconnect the colonic epithelial cells from connective tissue. Excess tissue was removed and centrifuge IEC containing solution at room temperature of 500g for 5 min. The supernatant was discarded and the cells were lysed for further study.

### Western Blot Analysis

For Western blot analysis, mouse peritoneal macrophages were pre-treated with DMSO or Munronoid I (50 μM) for 2 h, LPS incubated for 4 h, ATP (5 mM) treated for another 30 min. Then culture supernatants were collected for further studies and cells were washed by PBS twice and lysed in NP40 lysis buffer containing a 1× protease inhibitor mixture. Cell lysates were collected and the protein concentrations were equaled by an enhanced BCA protein assay kit (Beyotime Biotechnology, China). Equal protein concentrations were subjected to 6% – 15% SDS-PAGE gels, transferred onto PVDF membranes, incubated with 5% skim milk, and then with indicated primary antibodies overnight at 4°C, followed by secondary antibodies incubation for 2 h at room temperature. Signals were recorded with the FluorChem E (Cell Biosciences, Santa Clara, USA).

### Ubiquitination Assay

HEK293T cells were co-transfected with Flag-NLRP3, HA-Ubiquitin (HA-Ub), HA-K48 linked-Ubiquitin (HA-K48), or HA-K63 linked-Ubiquitin (HA-K63) plasmids. After 24 h, the cells were treated with DMSO or Munronoid I (50 μM) and MG132 (20 μM). After 5 h, the cells were collected and lysed in immunoprecipitation buffer (containing protease inhibitor cocktail and 1% SDS). The cell lysate was collected and a quarter of it was used as whole cell lysate (WCL). The remaining lysate was immunoprecipitated with anti-Flag antibody for 2 h, and then protein A/G Plus-Agarose immunoprecipitation reagent (Bimake, TX, USA) were added and shacked overnight at 4°C. The next day, immunoprecipitation buffer washed beads five times and protein was released by the SDS sample buffer. Immunoprecipitated sample and WCL were detected by Western blot analysis.

### Statistical Analysis

All data are presented as the mean ± SD, and at least three independent experiments were performed in duplicate. GraphPad Prism 6.0 was used for plotting data. The treatment effects between the two groups were statistically analyzed by Student’s t-test, and p < 0.05 was considered statistically significant.

## Results

### Munronoid I Treatment Alleviates DSS-Induced Colitis in Mice

The chemical structure of Munronoid I is shown in **Figure 1A**. Given the important role of Munronoid I in regulating inflammation, we investigated the potential therapeutic value of Munronoid I in colitis. DSS-induced colitis model is a well-established animal disease model, which is similar to clinical colitis in terms of symptoms and pathology, and this has great implications for drug development and disease research (53). The design of the experiment is shown in **Figure 1B**, and mice were treated with Munronoid I (10 mg/kg) for the duration of colitis induced by 2% or 3.5% DSS. The mice treated with DSS suffered severe body weight loss, consistent with weight changes, while Munronoid I mitigated the loss of body weight (**Figures 1C** and **S1A**). The disease activity index (DAI) scores were raised in DSS-treated mice, which were also decreased by Munronoid I treatment (**Figures 1D** and **S1B**). Shorter and erythematous colons, as key markers of colitis, were found in DSS-treated mice, which were rescued by Munronoid I (**Figures 1E, F**, **S1C, D**). Munronoid I treatment also reduced the inflammatory cytokines IL-6 and TNF-α production in colon tissues from DSS-induced mice (**Figures S1E, F, S2A, B**). In addition, compared with the DMSO+DSS group, histological examination of the colons in epithelial cell destruction and distortion of crypt structure were less damaged and inflammatory cell infiltration was reduced in the Munronoid I administration group (**Figures 1G, H**, **S2G, H**). The above results suggest that Munronoid I could ameliorate DSS-induced colitis.

### Munronoid I Reduced the Inflammasome Activation and Pyroptosis in Colon Tissues From DSS-Induced Mice

**Figure 2:**
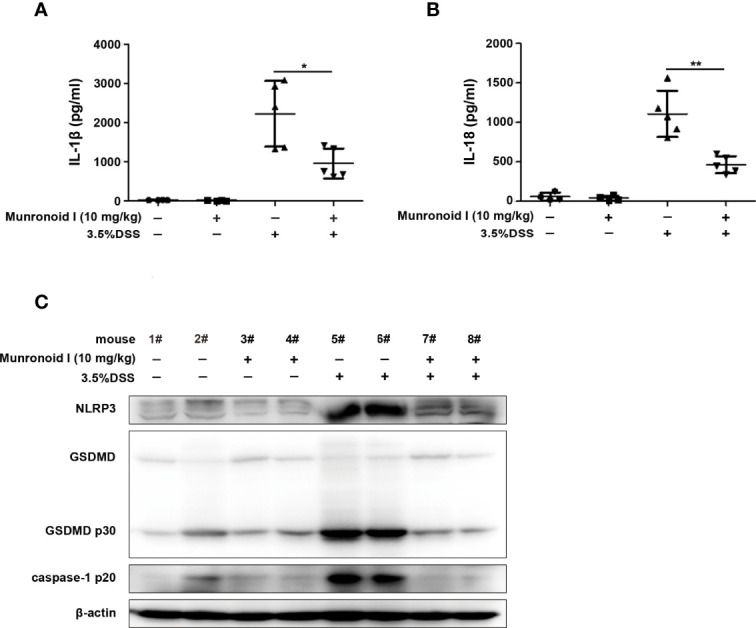
Munronoid I reduced the inflammasome activation and pyroptosis in colon tissues from DSS-induced colitis mice. **(A, B)** One piece of colon for ELISA to analyze production of cytokine IL-1β **(A)** and IL-18 **(B)**. **(C)**Western blot assay was used to detect pyroptosis related protein in the colon tissue. Data in A and B presented as the mean ± SD. *p<0.05, **p<0.01.

Previous reports demonstrated that the activity of NLRP3 inflammasomes were closely related to the development and pathogenesis of IBD and high expression and protein levels of pyroptosis-related factors in IBD patients (54). We further explored whether Munronoid I exerted its anti-colitis effect of Munronoid I through suppressing the NLRP3 inflammasome activation and pyroptosis. The production of inflammatory cytokines IL-1β and IL-18 in colon tissues were detected by ELISA. As shown in **Figures 2A, B**, **S3A, B**, Munronoid I treatment reduced the production of IL-1β and IL-18 in colon tissues from DSS-induced mice. Moreover, the expression of NLRP3, cleaved caspase-1, and pyroptosis-related protein cleaved GSDMD were detected by Western blot assay. The results showed that the protein expression of NLRP3, cleaved GSDMD (p30), and cleaved caspase-1 (p20) elevated in DSS-treated colon tissues were significantly reduced by Munronoid I administration (**Figures 2C** and **S3C**). These data indicate that Munronoid I could suppress inflammasome activation and pyroptosis in DSS-induced colitis.

### Munronoid I Suppresses LPS/ATP-Induced Caspase-1 Cleavage and IL-1β Maturation in Mouse Peritoneal Macrophages

**Figure 3:**
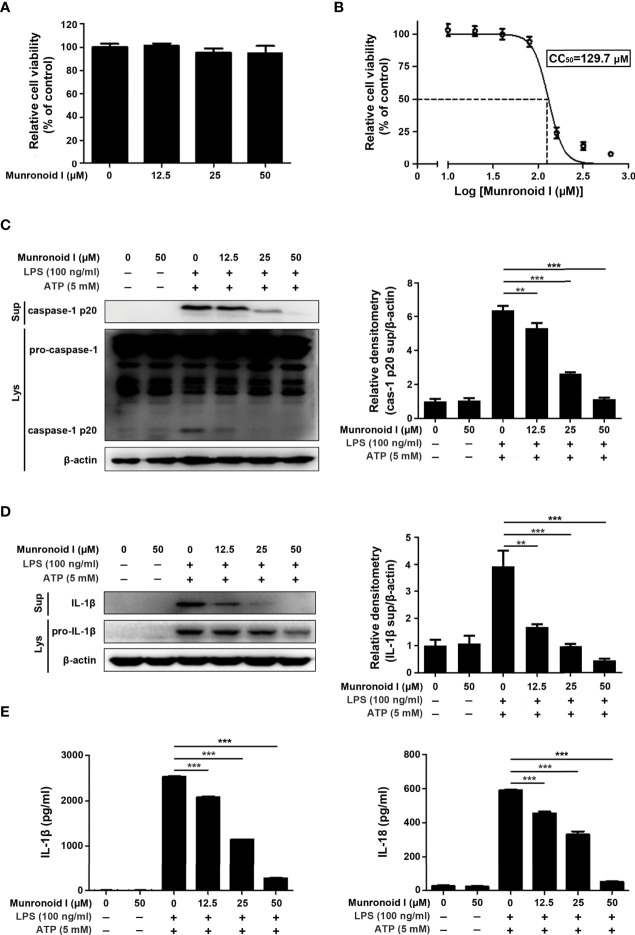
Munronoid I suppresses the secretion of pro-inflammatory cytokines in LPS/ATP-stimulated macrophages. **(A)** Mouse peritoneal macrophages were treated with DMSO or different concentrations of Munronoid I (12.5 μM, 25 μM, 50 μM) for 24 h, the cell viability was determined by CCK-8 assay. Results presented as mean ± SD (n=5). **(B)** Mouse peritoneal macrophages were pretreated with DMSO or different concentrations of Munronoid I (20 μM, 40 μM, 60 μM, 80 μM, 160 μM, 320 μM, or 640 μM) for 24 h, following detected by CCK-8 assay to calculated the CC50 of Munronoid **(I) (C, D)** Western blot was used to detect caspase-1 **(C)** and IL-1β **(D)** both in the cells’ lysate and supernatants, relative densitometry analyzed by Image **(J, E)** The supernatants of mouse peritoneal macrophages were collected and ELISA was employed to detect IL-1β and IL-18 production. Data in A, B, C, D, and E presented as the mean ± SD of three independent experiments. **p<0.01, ***p<0.001.

Macrophages are the main immune cells that contribute to the inflammatory response in the colon. So we chose mouse peritoneal macrophages for *in vitro* assays, and the potential cytotoxicity of Munronoid I was evaluated by the CCK-8 assay. Mouse peritoneal macrophages were incubated with Munronoid I at different concentrations (0, 12.5, 25, 50 μM) for 24 h. The data showed that the viability of macrophages was not affected by Munronoid I (**Figure 3A**). And CCK-8 assay was thus conducted to evaluate the median cytotoxic concentration (CC50) of test Munronoid I in concentrations ranging from 0 to 640 μM on mouse peritoneal macrophages. As shown in **Figure 3B**, the cytotoxic concentrations of Munronoid I that reduced the cell viability to 50% of the DMSO control (CC50) were calculated, and the CC50 value for the compound was 129.7 μM. Thus, non-cytotoxic concentrations of Munronoid I were used to treat mouse peritoneal macrophages in subsequent studies.

Since Munronoid I can attenuate DSS-induced colon injury and inflammatory cytokine production in mice, we further detected the anti-inflammatory effect of Munronoid I in mouse peritoneal macrophages. Caspase-1 plays an important role in the maturation of IL-1β and IL-18, and the Western blot results indicated that Munronoid I suppressed the expression of activated caspase-1 p20 subunit (**Figure 3C**) and IL-1β (**Figure 3D**). The supernatants with Munronoid I pretreatment and LPS/ATP stimulation were collected and analyzed by ELISA assay. The same results were verified by ELISA assay, Munronoid I decreased the production of inflammatory cytokines IL-1β and IL-18 (**Figure 3E**), and both IL-1β and IL-18 were reduced by up to 80% at high concentrations (50 μM) treatment.

### Munronoid I Inhibits the Activation of LPS/ATP-Induced Canonical Pyroptosis in Mouse Peritoneal Macrophages

**Figure 4:**
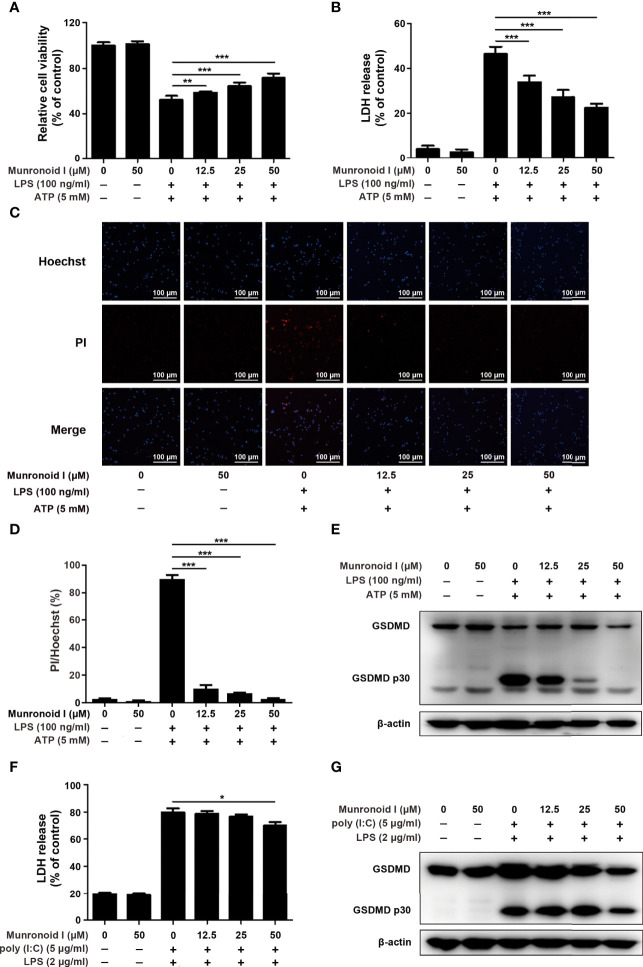
Munronoid I suppresses LPS/ATP-induced activation of canonical pyroptosis pathway. **(A, B)** Mouse peritoneal macrophages were pretreated with DMSO or Munronoid I (12.5 μM, 25 μM, 50 μM) for 2 h, following LPS (100 ng/ml) stimulation for 4 h, and then treated ATP (5 mM) for 30 min. And the cells viability was detected by CCK-8 assay **(A)**. The supernatants were analyzed by LDH assay **(B)**. **(C, D)** Mouse peritoneal macrophages were pretreated with DMSO or Munronoid I (12.5 μM, 25 μM, 50 μM) for 2 h, following LPS (100 ng/ml) stimulation for 4 h, and then treated ATP (5 mM) for 1 h. Hochest33342 and PI double staining images were captured by fluorescence microscopy (10×), merged with bright-field images **(C)**. The ratio of Hoechst and PI was analyzed by Image J **(D)**. And GSDMD and p30 subunit were detected by Western blot **(E)**. **(F, G)** Mouse BMDMs were pretreated with DMSO or Munronoid I (12.5 μM, 25 μM, 50 μM) for 2 h, following poly I:C (5 μg/ml) stimulation for 4 h, and then LPS (2 μg/ml) was transfected into the cells for 16 h. The supernatants were analyzed by LDH assay **(F)**. GSDMD were detected by Western blot **(G)**. Data in A, B, D, and F presented as the mean ± SD of three independent experiments. *p<0.05, **p<0.01, ***p<0.001.

Previous studies have indicated that LPS/ATP could induce canonical pyroptosis (55). To examine the effect of Munronoid I on LPS/ATP-induced pyroptosis, we pretreated mouse peritoneal macrophages with Munronoid I and stimulated them with LPS/ATP, then evaluated cell viability by CCK-8 assay. The results (**Figure 4A**) showed that the cell viability rate of 50% was increased to 70% with Muronoid I pre-treatment. LDH outflow through the GSDMD-pores, a large release of lactate dehydrogenase (LDH), is also a recognized feature of pyroptosis, so we further measured the effects of Munronoid I on the release of LDH with LPS/ATP stimulation. With the concentrations of Munronoid I increasing (**Figure 4B**) and the release of LDH gradually decreasing, 50μM Munronoid I has the most remarkable effect (about 50% inhibition ratio).

With the development of pyroptosis, the final result is the rupture of the cell membrane. Hochest33342 and PI double staining were used to detect the integrity of the cell membrane. Hochest33342 stained the nucleus blue and the cells with damaged membrane were stained red with PI. (**Figure 4C**). The analysis of relative cell viability by Image J illustrated that Munronoid I-pretreated groups reduced the number of damaged cells compared with positive control groups; Munronoid I almost restored the cell damage caused by LPS/ATP (**Figure 4D**) stimulation. GSDMD, as the executor of pyroptosis, activated p30 subunits cluster to form pores in the cell membrane (39, 40), which suggested that Munronoid I may have an inhibitory effect on the GSDMD p30 subunit. Then the GSDMD content was detected by Western blot assay. As we expected, Munronoid I reduced the production of the activated GSDMD p30 subunit (**Figure 4E** and **Figure S2A**). In conclusion, these findings indicated that Munronoid I inhibited LPS/ATP-induced pyroptosis in a dose-dependent manner.

LPS and ATP treatment could induce mouse peritoneal macrophage pyroptosis in the canonical pathway and intracellular LPS could stimulate the non-canonical pyroptosis pathway, which is NLRP3 inflammasome-independent (32). We wanted to further investigate the effect of Munronoid I on the non-canonical pathway, so the mouse bone marrow-derived macrophages (BMDMs) were transfected with LPS to induce non-canonical pyroptosis. BMDMs were pretreated with Munronoid I, incubated with poly I:C, and then transfected with LPS by FuGENE agent. LDH assay (**Figure 4F**) and CCK8 assay (the result is not shown) both demonstrated that Munronoid I did not relieve the intracellular LPS-induced non-canonical pyroptosis, with only a slight effect observed at high concentrations. Followed by detected GSDMD, the results illustrated that Munronoid I hardly repressed intracellular LPS induced the production of the activated GSDMD p30 subunit (**Figure 4G** and **Figure S2B**). Taking all of these data together, we suggest that Munronoid I significantly inhibited activation of the canonical pyroptosis pathway but only had a slight effect on the non-canonical pyroptosis pathway.

### Munronoid I Suppresses the Activation of LPS/ATP-Induced Canonical Pyroptosis in MODE-K Cells

**Figure 5:**
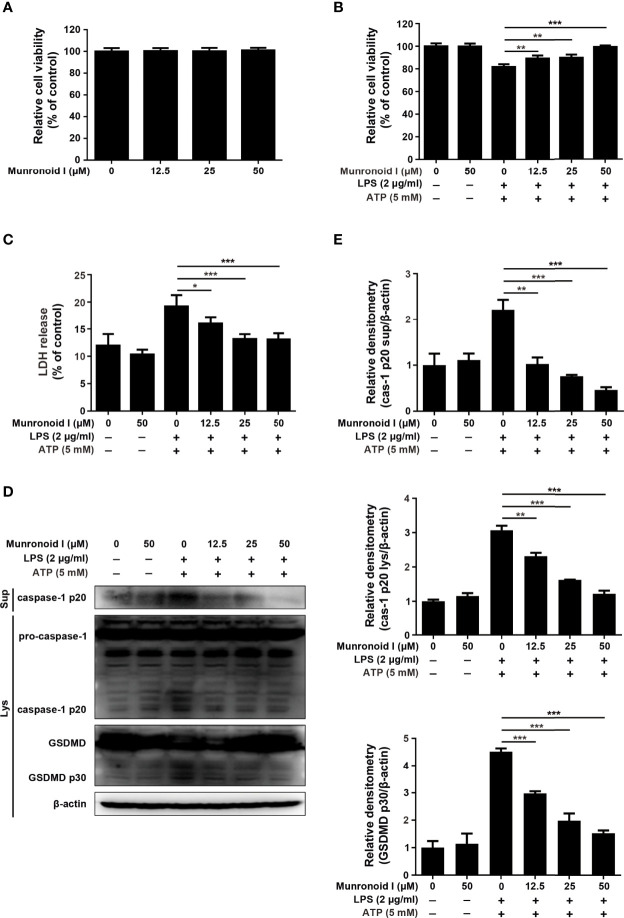
Munronoid I suppresses LPS/ATP-induced the activation of canonical pyroptosis pathway in MODE-K cells. **(A)** MODE-K cells were treated with DMSO or different concentrations of Munronoid I (12.5μM, 25μM, 50 μM) for 24 h, the cell viability was determined by CCK-8 assay. Results presented as mean ± SD (n=5). **(B–D)** MODE-K cells were pretreated with DMSO or Munronoid I (12.5 μM, 25 μM, 50 μM) for 2 h, following LPS (2 μg/ml) stimulation for 4 h, and then treated ATP (5 mM) for 30 min. And the cells viability was detected by CCK-8 assay **(B)**. The supernatants were analyzed by LDH assay **(C)**. And caspase-1 and GSDMD were detected by Western blot **(D)**. Relative densitometry analyzed by Image J **(E)**. Data in A, B, C, and E presented as the mean ± SD of three independent experiments. *p<0.05, **p<0.01, ***p<0.001.

Previous data have revealed that Munronoid I inhibited LPS/ATP-induced the canonical pyroptosis in mouse peritoneal macrophages, and some studies proved that the NLRP3 inflammasome plays an important role in the occurrence and development of IBD (36–38). First, the CCK-8 assay (**Figure 5A**) determined that Munronoid I had no apparent toxicity to the mouse intestinal epithelial cell line MODE-K. Then we used LPS and ATP to induce pyroptosis in MODE-K cells, the CCK-8 assay (**Figure 5B**) and the LDH assay (**Figure 5C**) both showed that we succeeded in inducing pyroptosis in MODE-K cells and Munronoid I could alleviate the mode of cell death. As shown in **Figures 5D, E**, we obtained consistent results as in mouse peritoneal macrophages, the protein level of caspase-1 p20 and GSDMD p30 decreased with the increasing concentrations of Munronoid I in MODE-K cells.

### Munronoid I Suppresses LPS/ATP-Induced Pyroptosis *via* Regulating NLRP3 Expression

**Figure 6:**
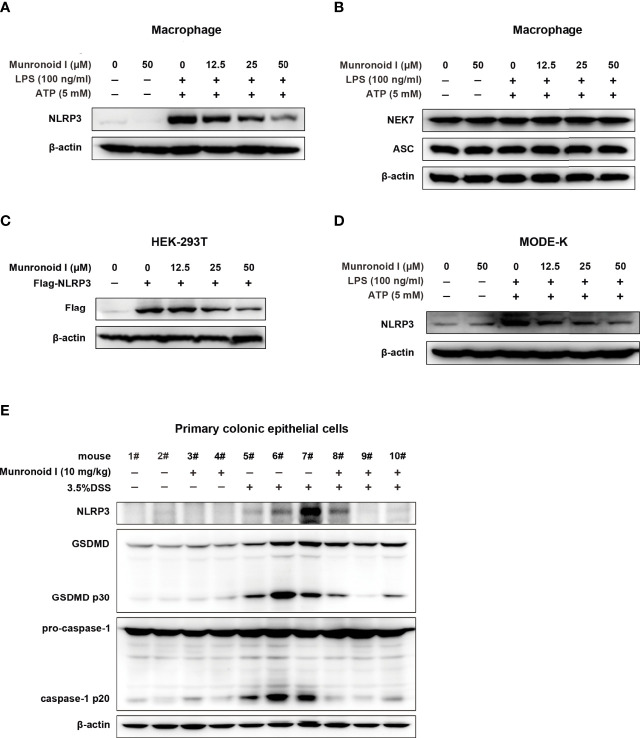
Munronoid I downregulated the protein level of NLRP3 in macrophages and MODE-K cells stimulated with LPS/ATP. **(A, B)** Mouse peritoneal macrophages were pretreated with DMSO or different concentrations of Munronoid I (12.5 μM, 25 μM, 50 μM) for 2 h, following stimulation with LPS (100 ng/ml) for 4 h, and then treated with ATP (5 mM) for 30 min. The cell lysate was collected and Western blot was performed to detect NLRP3 **(A)**, NEK7 and ASC **(B)**. **(C)** Plasmid Flag-NLRP3 was transfected into HEK293T cells. DMSO or different concentrations of Munronoid I (12.5 μM, 25 μM, 50 μM) were added the cells for 4 h, Western blot analysis of NLRP3 protein in cell lysates with antibody to Flag tag. **(D)** MODE-K cells were pretreated with DMSO or Munronoid I (12.5 μM, 25 μM, 50 μM) for 2 h, following LPS (2 μg/ml) stimulation for 4 h, and then treated with ATP (5 mM) for 30 min, NLRP3 protein were analyzed by Western blot. All the above data were from three independent experiments. **(E)** C57BL/6 mice (n = 4–5 mice/group) were fed with distilled water or water containing 3.5% DSS and were intragastric injected with DMSO or Munronoid I (10 mg/kg) daily. Seven days later, all the water was changed to distilled water, on the 8th day, the mice were sacrificed and their colonic epithelial cells were collected and Western blot was performed to detect NLRP3, caspase-1 and GSDMD.

The NLRP3 inflammasome is a multiple proteins complex that consists of NLRP3, ASC, pro-caspase-1, and NIMA-related kinase 7 (NEK7). It is essential to the development of canonical pyroptosis. We wondered whether Munronoid I might inhibit the production of NLRP3, ASC, pro-caspase-1, or NEK7 to suppress pyroptosis, so we detected the proteins extracted from the Munronoid I-treated mouse peritoneal macrophages. The Western blot results indicated that Munronoid I suppressed the production of activated caspase-1 p20 subunit (**Figure 3C**) and NLRP3 (**Figure 6A**), but Munronoid I has little effect on pro-caspase-1, ASC, and NEK7 (**Figures 3C**, **6B**). The assembly of NLRP3 inflammasome plays an essential role in the maturation of caspase-1. We, therefore, suggest that inhibition of NLRP3 production by Munronoid I leads to inhibition of pro-caspase-1 cleavage. To verify our hypothesis, HEK293T cells with plasmid overexpressing Flag-NLRP3 plasmid were treated with Munronoid I at different concentrations, and we found that the protein level of NLRP3 was negatively correlated with the concentrations of Munronoid I (**Figure 6C**). The consistent result was also found in MODE-K cells (**Figure 6D**). Then we extracted the primary colonic epithelial cells from the mice treated with 3.5%DSS and found that Munronoid I decreased NLRP3 expression (**Figure 6E**). The western blot assay results indicated that Munronoid I suppressed LPS/ATP-induced pyroptosis *via* inhibiting NLRP3 expression.

### Munronoid I Promotes K48-Linked Ubiquitination and Degradation of NLRP3

**Figure 7:**
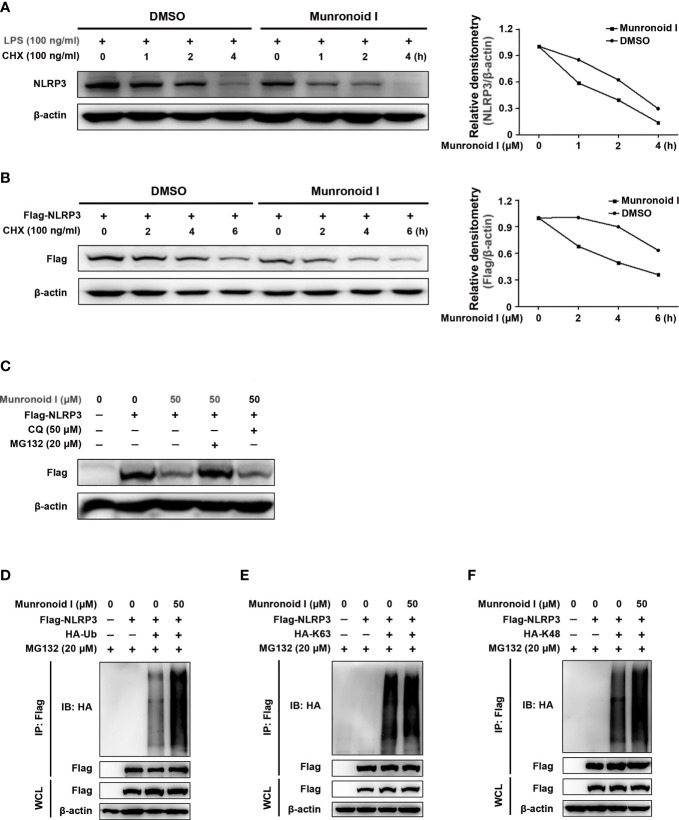
Munronoid I promotes the K48-linked ubiquitination and proteasomal degradation of NLRP3. **(A)** Mouse peritoneal macrophages were treated with DMSO or Munronoid I (50 μM) for 4 h and stimulated with CHX (100 ng/ml) for indicated hours, following NLRP3 was detected by Western blot. **(B)** Plasmid encoding Flag-NLRP3 was transfected into HEK293T cells. The cells were treated with DMSO or Munronoid I (50 μM) for 6 h and stimulated with CHX (100 ng/ml) for indicated hours, following Flag were detected by Western blot. All the above data were from three independent experiments. **(C)** Plasmid encoding Flag-NLRP3 was transfected into HEK293T cells. The cells were treated with DMSO or Munronoid I (50 μM) and MG132 (20 μM) or CQ (50 μM) for 5 h, Flag was detected by Western blot. **(D–F)** Plasmids encoding Flag-NLRP3 and HA-Ub/HA-K48/HA-K63 were co-transfected into HEK293T cells for 24 h. Cells were treated with DMSO or Munronoid I (50 μM) and MG132 (20 μM) for 5 h. Cells were harvested for Western blot analysis of HA-Ub **(D)** or HA-K63 **(E)** or HA-K48 **(F)** immunoprecipitated with antibody to Flag tag. All the above data were from three independent experiments.

To further determine the pathway involved in the reduction of NLRP3 content by Munronoid I, protein synthesis inhibitor CHX and protein degradation inhibitors MG132 or CQ were employed to treat mouse peritoneal macrophages or NLRP3-overexpressed HEK293T cells pretreated or not by Munronoid I. As shown in **Figures 7A, B**, Munronoid I could promote NLRP3 degradation and even inhibit its protein synthesis. To determine the effect of Munronoid I on degradation of NLRP3, we used proteasome inhibitor MG132 and autophagy inhibitor CQ to treat NLRP3-overexpressed HEK293T cells to identify the pathway involved in NLRP3 degradation. The result showed that MG132 could block Munronoid I-induced NLRP3 degradation (**Figure 7C**), which meant that Munronoid I promotes the degradation of NLRP3 by the proteasome pathway. A previous study has illustrated that proteins modified by ubiquitination are a marker of proteasomal degradation (56), so HEK293T cells co-transfected with Flag-NLRP3 plasmid and HA-Ub plasmid following MG132 and Munronoid I treatment. We found that Munronoid I increased the ubiquitination level of NLRP3 (**Figure 7D**). Further experimental results showed that Munronoid I had no effect on the K63-linked ubiquitination (**Figure 7E**), but significantly increased the Lys48 (K48)-linked ubiquitination of NLRP3 (**Figure 7F**). Taking all of these experimental data together, it is demonstrated that Munronoid I promoted the K48-linked ubiquitination-dependent proteasomal degradation of NLRP3, which disrupted the assembly of NLRP3 inflammasome to implement the effect of inhibiting cell pyroptosis.

## Discussion

Herein, we successfully established a cellular canonical and non-canonical pyroptosis model *in vitro* and a DSS-induced colitis model *in vivo*. Munronoid I remarkably inhibited ATP/ LPS-induced canonical pyroptosis in mouse peritoneal macrophages but had little effect on intracellular LPS-induced non-canonical pyroptosis in BMDMs, and attenuated DSS-induced colitis *in vivo*. Regarding the underlying mechanism, NLRP3 is essential for canonical pyroptosis activation, but the non-canonical pathway is independent of NLRP3. We used protein synthesis inhibitor CHX, proteasome inhibitor MG132, and autophagy inhibitor CQ to demonstrate that Munronoid I increased the NLRP3 degradation *via* the proteasome. As revealed by ubiquitination assays, Munronoid I might suppress the canonical pyroptosis by promoting K48-linked ubiquitination of NLRP3.

Cell death is necessary for most physiological processes and was once believed to be two distinct processes; apoptosis and necrosis. Several new types of cell death have been recently discovered, one of which is pyroptosis. Unlike apoptosis and necrosis, pyroptosis is an immune defense response to pathogen invasion and is accompanied by plasma membrane disruption and inflammatory cytokine secretion, which is designed to eliminate pathogens (57–59). Overactive inflammasome-induced uncontrollable pyroptosis may cause the cell membrane destruction and massive efflux of inflammatory factors, disrupting homeostasis (28, 32, 39) and therefore resulting in the occurrence of some diseases. NLRP3 inflammasome has been reported to be associated with IBD, but the role of NLRP3 in IBD is not yet fully clarified as it appears to have both pathogenic and protective effects (37, 38, 60). NLRP3^-/-^ mice were highly susceptible to DSS-induced IBD and DSS-induced colitis-associated colorectal cancer (CAC) (37, 61), but DSS-induced IBD in WT mice was relieved *via* suppressing the expression of NLRP3 (38). These studies demonstrated that NLRP3 might play a role in the regulation of intestinal homeostasis, however, its overactivation and depletion may exaggerate the development of the disease in the case of colitis. Therefore, NLRP3 has become an attractive target for the development of therapies for IBD.

In innate immune cells, especially in macrophages, NLRP3 acts as a cytosolic pattern recognition receptor (PRR), suggesting that it not only mediates immune responses to foreign molecules but also responds to endogenous danger signals (62). NLRP3 inflammasome activation requires two signals; a priming signal and an activation signal. The first signal, such as LPS, induces the transcription and translation of NLRP3 *via* the NF-κB pathway, and then NLRP3 inflammasome activation could be induced by the second phase of stimuli, including ATP (55), the outflow of intracellular potassium (63), reactive oxygen species (ROS) (64), some crystal structure irritants like asbestos, silica, monosodium urate (MSU) (65–67), and the metabolites (68, 69). The inflammasome complex contains three components, NLRP3, ASC, and pro-caspase-1, and its activation subsequently initiates pyroptosis (23). Our previous study has proven that Munronoid I downregulate TAK1 and NF-κB signaling pathways, which are the upstream of NLRP3 expression, so Munronoid I can inhibit NLRP3 mRNA levels by TAK1 and NF-κB pathway weakly (the result is not shown). Our results indicated that Munronoid I downregulated the NLRP3 protein level in independent TAK1 and NF-κB pathways, but had no effect on ASC and pro-caspase-1, which in turn inhibited the production of caspase-1 p20 and GSDMD p30 subunits. Three other inflammasomes NLR family pyrin domain-containing 1 (NLRP1), absent in melanoma 2 (AIM2), and NLR-family CARD-containing protein 4 (NLRC4) have been reported to be involved in the recruitment of the adaptor protein ASC, the cleavage of caspase-1, and the secretion of mature IL-1β and IL-18 proteins (70). Our data showed that Munronoid I inhibited NLRP3 inflammasome activation. And the macrophages were treated by flagellin or poly(dA:dT), Munronoid I could not decrease the production of IL-1β in macrophages (the result is not shown), so Munronoid I might be specific to NLRP3 inflammasome. Further studies are also needed to determine if this molecule has any effect on the other inflammasome.

The posttranslational modification ubiquitination plays a significant role in modulating the NLRP3 inflammasome signaling pathway. Both K48-linked and K63-linked ubiquitination of NLRP3 negatively regulates its activation. F box protein, F-box L2 (FBXL2), recognizes and interacts with Trp-73 of NLRP3 and targets Lys-689 within NALP3 for ubiquitination and proteasomal degradation (71). Tripartite motif-containing protein 31 (TRIM31) (72) and membrane-associated RING-CH-type finger 7 (MARCH7) (73) promote K48-linked ubiquitin ligation of NLRP3; the former induces NLRP3 degradation through the proteasomal pathway, while the latter induces NLRP3 degradation through the autophagy pathway. Ariadne homolog 2 (ARIH2) interacted with the NACHT domain of NLRP3 and downregulated NLRP3 inflammasome activity through NLRP3 K48- and K63-linked ubiquitination (74). The deubiquitination enzyme BRCA1/BRCA2-containing complex subunit 3 (BRCC3) is a positive regulator of NLRP3 activity *via* promoting NLRP3 K63-linked deubiquitination (75, 76). The stimulator of interferon genes (STING) interacts with NLRP3 and attenuates K48- and K63-linked polyubiquitination of NLRP3, thereby promoting the inflammasome activation (77). Munronoid I could increase the K48-linked ubiquitination of NLRP3 but has little effect on the K63-linked ubiquitination of NLRP3. Our previous study has demonstrated that Munronoid I could promote the TAK1 K48-linked polyubiquitination, thus, further study is needed to detect whether Munronoid I targets a certain protein to promote degradation of NLRP3 and TAK1.

In conclusion, LPS/ATP-induced NLRP3 inflammasome activation leads to an increase in cell pyroptosis. Munronoid I promoted K48-linked ubiquitination and degradation of NLRP3, therefore modulating the production of pro-inflammatory factors and the canonical pyroptosis in mouse peritoneal macrophages and DSS-induced IBD in mice. Consequently, Munronoid I may be a therapeutic candidate for the treatment of IBD or NLRP3-related disease.

## Data Availability Statement

The original contributions presented in the study are included in the article/**Supplementary Material**. Further inquiries can be directed to the corresponding author.

## Ethics Statement

The animal study was reviewed and approved by the Animal Care and Use Committee of Shenzhen University School of Medicine (Approval Number: AEWC-2019011).

## Author Contributions

WC, WX, and XM performed study concept and design; XL extracted and purified Munronoid I from Munronia sinica. XM, QD, XZ, YX, and XL provided acquisition, analysis, and interpretation of data, and statistical analysis. ZW, HW, RZ, HT, and JQ provided technical and material support. XM and WC developed the methodology and undertook writing, review, and revision of the paper. All authors read and approved the final paper.

## Funding

This work was supported by grants from the National Natural Science Foundation of China (No. U1801283, 31870908, 81903541), a Joint project from Yunnan Science and Technology Office and Yunnan University (No.2018FY001-001), SZU Top Ranking Project (No.86000000210) to Weilin Chen, and Project of Innovative Research Team of Yunnan Province (202005AE160005) to WX

## Conflict of Interest

The authors declare that the research was conducted in the absence of any commercial or financial relationships that could be construed as a potential conflict of interest.

## Publisher’s Note

All claims expressed in this article are solely those of the authors and do not necessarily represent those of their affiliated organizations, or those of the publisher, the editors and the reviewers. Any product that may be evaluated in this article, or claim that may be made by its manufacturer, is not guaranteed or endorsed by the publisher.

## Abbreviations

IBD, Inflammatory bowel diseases; CD, Crohn’s disease; UC, ulcerative colitis; caspase, cysteine-dependent aspartate-specific proteases; LPS, lipopolysaccharide; ATP, adenosine triphosphate; NLRP3, NOD-like receptor pyrin domain-containing protein 3; DSS, Dextran sulfate sodium salt; LRR, C-terminal leucine-rich repeat domain; NACHT, nucleotide binding and oligomerization domain; PYD, domain, pyrin domain; IL-1β, Interleukin 1 beta; GSDMD, gasdermin D; GSDMD p20, GSDMD C-terminal repressor domain; GSDMD p30, GSDMD N-terminal pore-forming domain; DAI, disease activity index; LDH, lactate dehydrogenase; CC50, median cytotoxic concentration; K48, Lys48; PRR, pattern recognition receptor; ROS, reactive oxygen species; MSU, monosodium urate; NLRP1, NLR family pyrin domain-containing 1; AIM2, absent in melanoma 2; NLRC4, NLR-family CARD-containing protein 4; FBXL2, F box protein, F-box L2; TRIM31, tripartite motif-containing protein 31; MARCH7, membrane-associated RING-CH-type finger 7; ARIH2, Ariadne homolog 2; BRCC3, BRCA1/BRCA2-containing complex subunit 3; STING, stimulator of interferon genes.
