# Supplementary material for: Munronoid I Ameliorates DSS-Induced Mouse Colitis by Inhibiting NLRP3 Inflammasome Activation and Pyroptosis Via Modulation of NLRP3
Source: Front Immunol. 2022 Jul 5;13:853194. doi: 10.3389/fimmu.2022.853194 (PMC9296101; doi:10.3389/fimmu.2022.853194)
Supplement: Supplementary file 1 [file DataSheet_1.docx]

**Supplementary Information:**

**Munronoid I ameliorates DSS-induced mouse colitis by inhibiting NLRP3 inflammasome activation and pyroptosis via modulation of NLRP3**

**Xingyu Ma^1 #^, Qianqian Di^1#^, Xiaoli Li^2^, Qianqian Di^1^, Xibao Zhao^1^, Ruihan Zhang^2^, Yue Xiao^1^, Xunwei Li^1^, Han Wu^1^, Haimei Tang^1^, Jiazheng Quan^1^, Zherui Wu^1^, Weilie Xiao^2*^ Weilin Chen^1*^**

^1^Marshall Laboratory of Biomedical Engineering, Department of Immunology, Shenzhen University School of Medicine, Shenzhen, 518060, China

^2^Key Laboratory of Medicinal Chemistry for Natural Resource, Ministry of Education, Yunnan Research & Development Center for Natural Products, School of Chemical Science and Technology, Yunnan University, Kunming 650091, China

**^#^** These authors contributed equally.

**
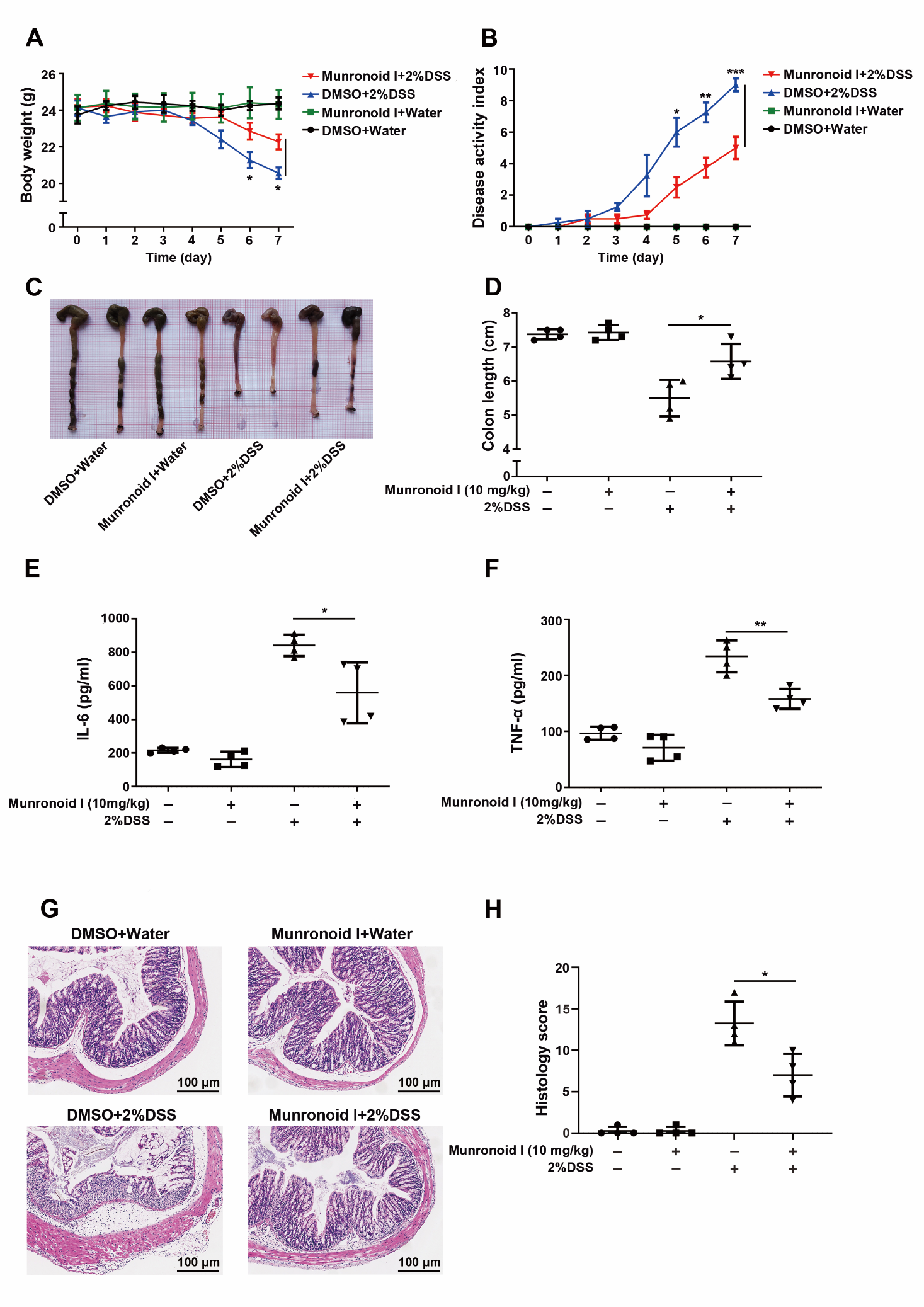
**

**Figure S1.** C57BL/6 mice (n = 4 mice/group) were fed with distilled water or water containing 2% DSS and were intragastric injected with DMSO or Munronoid I (10 mg/kg) daily. Seven days later, all the water was changed to distilled water, on the eighth day, the mice were sacrificed and their colon tissue were collected. The body weight (A), stool consistence and gross rectal bleeding were recorded every day to calculate the DAI score (B). (C) Colons of mice were assessed at the time of necropsy with representative macroscopic images. (D) The colon length was measured and statistical significance determined by unpaired Student’s t test. (E-F) One piece of colons for ELISA to detect analyze production of cytokine IL-6 (E) and TNF-α (F). (G) H&E staining of colon sections (10X) and representative H&E images scored in (H). Data in A, B, D, E, F and H presented as the mean ± SD. *p<0.05, **p<0.01, ***p<0.001.


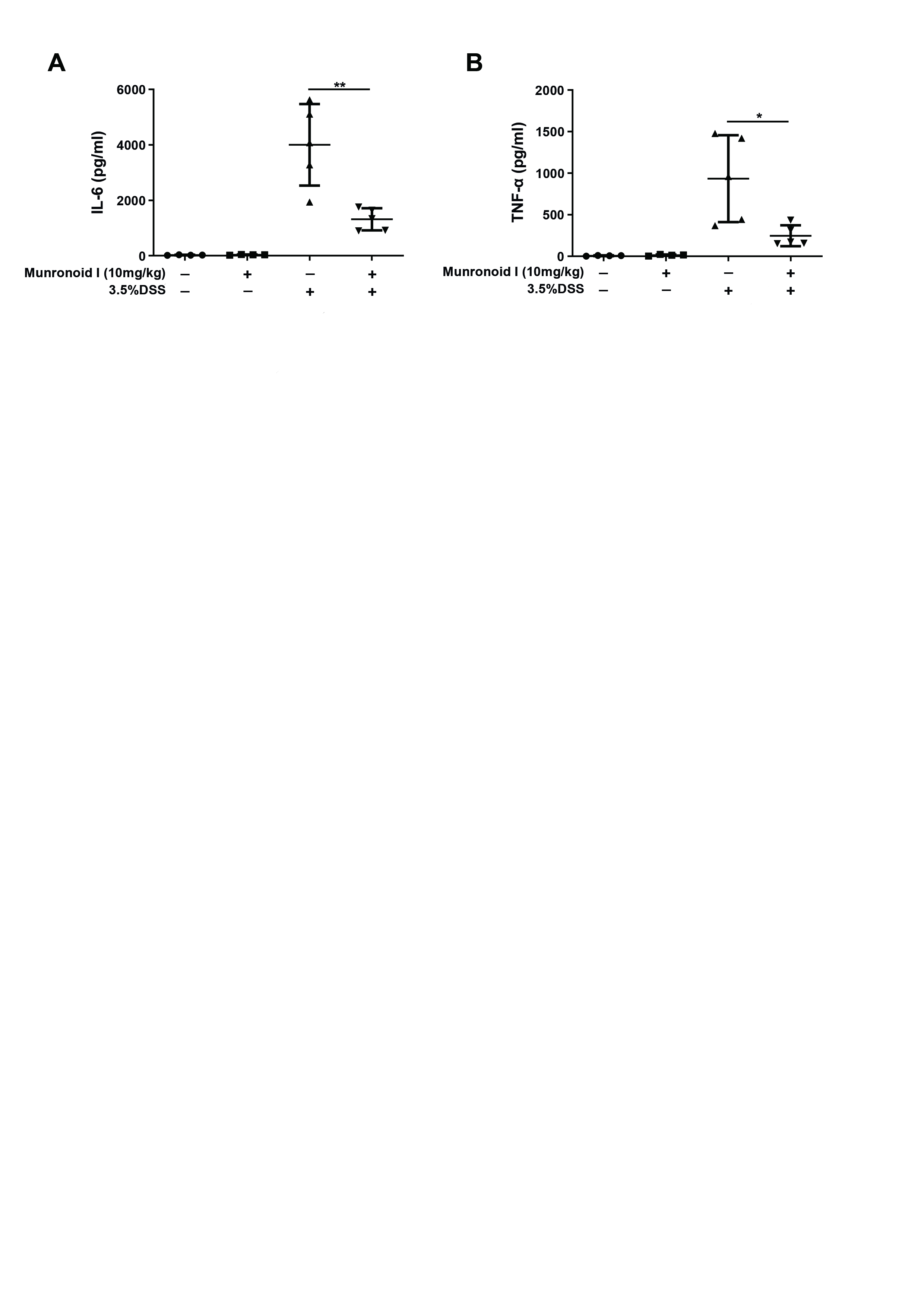


**Figure S2.** C57BL/6 mice (n = 4–5 mice/group) were fed with distilled water or water containing 3.5% DSS and were intragastric injected with DMSO or Munronoid I (10 mg/kg) daily. Seven days later, all the water was changed to distilled water, on the eighth day, the mice were sacrificed and their colon tissue were collected. (A-B) One piece of colons for ELISA to detect analyze production of cytokine IL-6 (A) and TNF-α (B). Data in A and B presented as the mean ± SD. *p<0.05, **p<0.01.


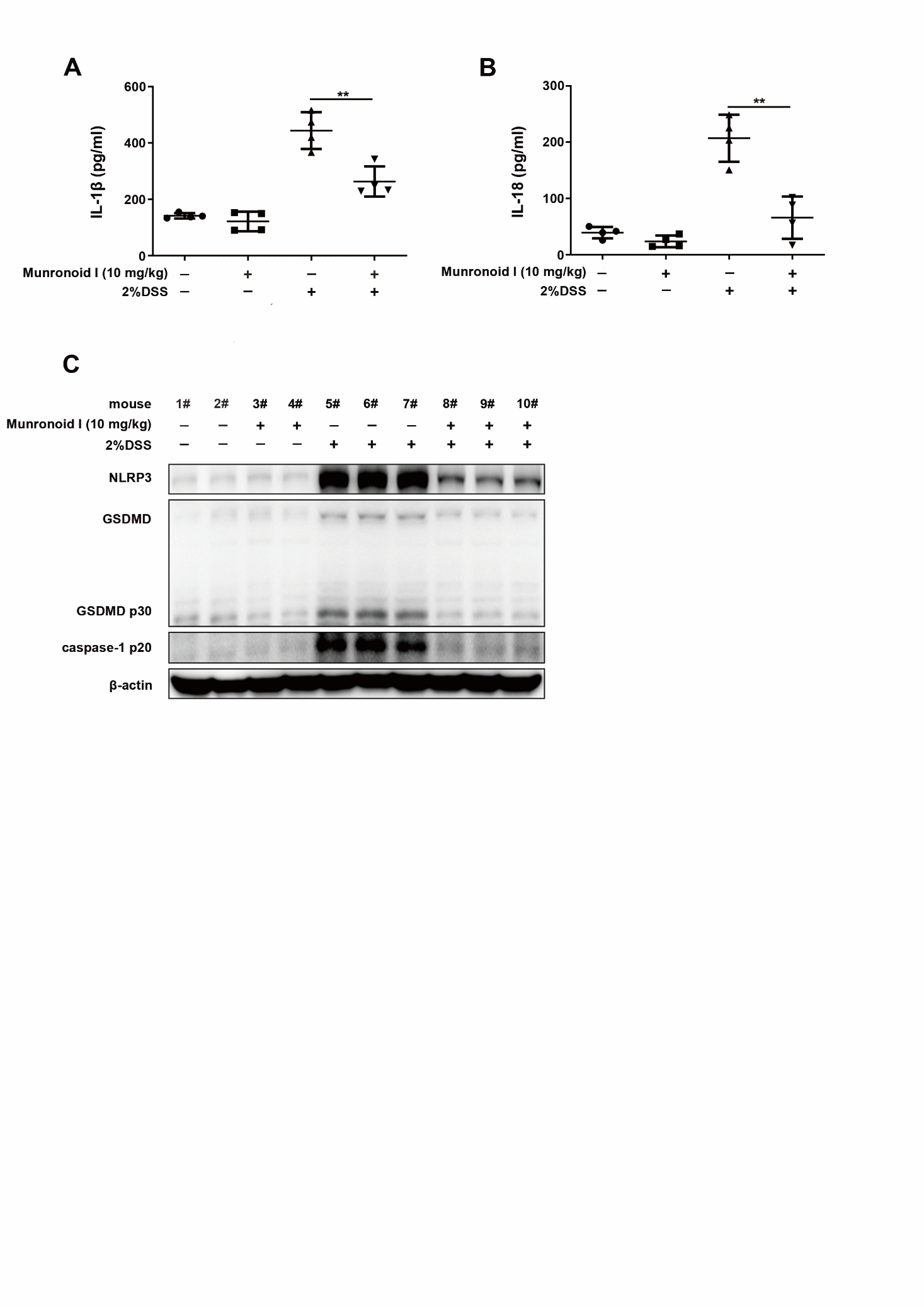


**Figure S3.** (A-B) C57BL/6 mice (n = 4 mice/group) were fed with distilled water or water containing 2% DSS and were intragastric injected with DMSO or Munronoid I (10 mg/kg) daily. Seven days later, all the water was changed to distilled water, on the eighth day, the mice were sacrificed and their colon tissue were collected. One piece of colons for ELISA to detect analyze production of cytokine IL-1β (A) and IL-18 (B). (C)Western blot assay was used to detect pyroptosis related protein in the colon tissue. Data in A and B presented as the mean ± SD. **p<0.01.

**
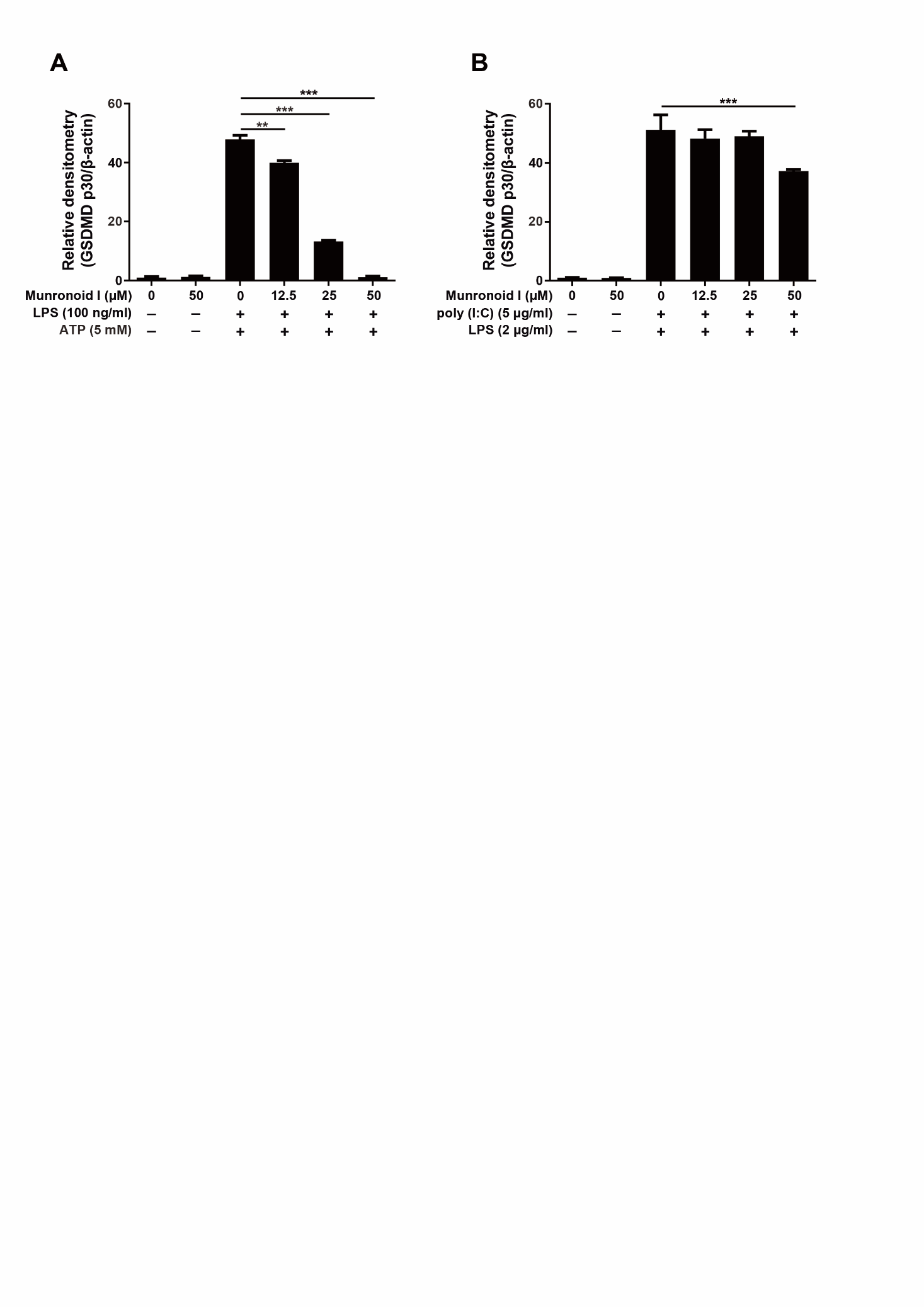
**

**Figure S4.**  (A) Mouse peritoneal macrophages were pretreated with DMSO or Munronoid I (12.5 μM, 25 μM, 50 μM) for 2 h, following LPS (100 ng/ml) stimulation for 4 h, and then treated ATP (5 mM) for 30 min. And GSDMD and p30 subunit were detected by Western blot, relative densitometry analyzed by Image J. (B) Mouse BMDMs were pretreated with DMSO or Munronoid I (12.5 μM, 25 μM, 50 μM) for 2 h, following poly I:C (5 μg/ml) stimulation for 4 h, and then LPS (2 μg/ml) was transfected into the cells for 16 h. GSDMD were detected by western blot, relative densitometry analyzed by Image J. Data in A and B presented as the mean ± SD. **p<0.01, ***p<0.001.
